# Neuroprotective Effects of Distilled Extract of *Zanthoxylum piperitum* in Parkinson’s Disease Models

**DOI:** 10.3390/nu18142350

**Published:** 2026-07-17

**Authors:** Su Bin Park, Jihun Gong, Gabsik Yang, Ye-eun Baek, Amjad Khan, Tae Han Yook, Ji Yong Jang, Jong Uk Kim

**Affiliations:** 1 Lee Gil Ya Cancer and Diabetes Institute, Gachon University, Incheon 21999, Republic of Korea; sws10089460@gmail.com (S.B.P.); yanggs@gachon.ac.kr (G.Y.); 2 College of Korean Medicine, Gachon University, Seongnam 13120, Republic of Korea; amjadkhan1901@gmail.com; 3 College of Korean Medicine, Woosuk University, Jeonju 54986, Republic of Korea; jhkong1022@gmail.com (J.G.); nasiss@naver.com (T.H.Y.); 4 Department of Food Regulatory Sciences, Korea University, Sejong 30019, Republic of Korea; rainbye23@gmail.com

**Keywords:** *Zanthoxylum piperitum*, Parkinson’s disease, neuroprotection

## Abstract

**Background**: Parkinson’s disease (PD) is a progressive neurodegenerative disorder characterized by the selective loss of dopaminergic neurons in the substantia nigra. Oxidative stress, neuroinflammation, and α-synuclein aggregation are central pathological features of PD. *Zanthoxylum piperitum* DC, commonly known as Korean pepper or chopi, is a traditional dietary spice in Eastern Asia and has been reported to possess antioxidant and anti-inflammatory properties. This study investigated the neuroprotective and motor function–enhancing effects of distilled extract of *Z. piperitum* (deZP) in 1-Methyl-4-phenylpyridinium (MPP^+^)-treated *Caenorhabditis elegans* and 1-methyl-4-phenyl-1,2,3,6-tetrahydropyridine (MPTP)-induced mouse models of PD. **Methods**: In the *C. elegans* model, dopaminergic neurotoxicity was induced by MPP^+^, and deZP was tested at 0.25, 0.5, and 1% (*v*/*v*) to evaluate neuronal preservation through GFP-labeled dopaminergic neurons and α-synuclein expression. Concurrently, in the MPTP-induced mouse model, deZP was administered intranasally at a fixed dose of 20 μL/mouse, equivalent to 5 mg/mouse. Motor function was assessed using the rota-rod test, pole test, and grip strength test, while dopaminergic neuronal survival was evaluated by tyrosine hydroxylase (TH) immunostaining. **Results**: In MPP^+^-treated *C. elegans*, deZP significantly restored green fluorescent protein (GFP) fluorescence in dopaminergic neurons and reduced α-synuclein expression, with the most pronounced effects observed at 1% (*v*/*v*). In the MPTP-induced mouse model, deZP at this fixed intranasal dose significantly improved motor performance and preserved TH-positive neurons in the substantia nigra. **Conclusions**: These findings suggest that deZP may represent a promising preclinical candidate for further investigation in PD-related neurodegeneration.

## 1. Introduction

Parkinson’s disease (PD) is a representative neurodegenerative disorder characterized by the selective degeneration of dopaminergic neurons in the substantia nigra (SNpc) of the midbrain [1]. Dopaminergic neurons in the SNpc project to the striatum and deliver dopamine, thereby playing essential roles in the initiation and regulation of movement, balance, and motor coordination [2,3]. Accordingly, damage to these neurons reduces dopamine levels in the nigrostriatal pathway, leading to cardinal motor symptoms such as bradykinesia, rigidity, resting tremor, and postural instability [3]. In addition to motor symptoms, PD is also associated with various non-motor symptoms, including cognitive impairment, depression, and sleep disturbances [4].

The pathogenesis of PD involves multiple interconnected mechanisms, including α-synuclein accumulation, mitochondrial dysfunction, neuroinflammation, and dopaminergic neuronal damage [5,6]. α-Synuclein is normally localized at presynaptic terminals, where it contributes to the maintenance of synaptic function by regulating synaptic vesicle trafficking and neurotransmitter release [7,8]. In PD, however, α-synuclein undergoes abnormal misfolding and aggregation, resulting in the formation of Lewy bodies [9]. These pathological changes are associated with mitochondrial dysfunction, impaired protein homeostasis, and neuroinflammatory responses [10,11]. In particular, aggregated α-synuclein is known to activate microglia, thereby promoting inflammatory responses and further exacerbating dopaminergic neuronal degeneration [12].

*Zanthoxylum piperitum* DC (Japanese pepper or Korean chopi) is an edible plant widely consumed as a culinary spice and seasoning throughout East Asia. In addition to its traditional use as a food ingredient, it has long been utilized in traditional medicine for the management of gastrointestinal disorders, pain, and inflammatory diseases [13]. Previous studies have reported that *Z. piperitum* or *Zanthoxylum*-derived materials may alleviate inflammatory and pain-related responses [14]. In addition, xanthoxyline, one of the constituents derived from *Z. piperitum*, has been reported to suppress dopaminergic neurodegeneration in a DAF-16-dependent manner in a *C. elegans* model [15]. Skimmianine, which is also found in plants of the genus *Zanthoxylum*, has been suggested to be associated with the regulation of microglial activation and neuroprotective effects [16]. These findings support the potential applicability of *Z. piperitum* and *Zanthoxylum*-derived materials in neurological disorders.

Based on these findings, the present study investigated whether a distilled extract of *Z. piperitum* (deZP) could exert neuroprotective effects against dopaminergic neurodegeneration. Previous phytochemical and pharmacokinetic profiling of this specific extract identified terpinen-4-ol as its major active component, along with other volatile constituents such as d-limonene, citronellal, and geraniol [17]. Among these constituents, terpinen-4-ol, d-limonene, citronellal, and geraniol have each been reported to exert antioxidant, anti-inflammatory, and neuroprotective activities, supporting their potential as food-derived bioactive compounds [18,19,20,21]. Notably, previous studies have demonstrated that deZP possesses a favorable safety profile, showing rapid systemic elimination without inducing significant acute toxicity or biochemical abnormalities in visceral organs in vivo [17]. This established tolerability and its distinct biochemical properties warrant further evaluation of its therapeutic potential against neurodegenerative disorders.

While this distilled extract is commercially manufactured for pharmacopuncture, the present study focused on evaluating the biological activity of its food-derived volatile phytochemicals. Therefore, this study investigated whether a deZP, rich in naturally occurring volatile bioactive compounds, could protect dopaminergic neurons and alleviate motor dysfunction in experimental models of PD. The intranasal route was selected because the formulation is a distilled aqueous preparation that can be delivered in a small volume, making it suitable for exploratory preclinical administration via the nasal route. In addition, intranasal delivery was considered relevant as a non-invasive route for evaluating central nervous system (CNS)-oriented biological effects while avoiding gastrointestinal degradation and first-pass metabolism [22,23,24,25]. Although intranasal administration was employed to facilitate proof-of-concept evaluation of central neuroprotective efficacy [26], the findings may provide a scientific basis for the future development of *Z. piperitum*-derived nutraceuticals and functional food ingredients targeting neurodegenerative disorders.

## 2. Materials and Methods

### 2.1. Materials

The deZP was purchased from AJ Pharmacopuncture (Seoul, Republic of Korea). According to the previously reported preparation method, deZP was produced by mixing the plant material with distilled water, followed by boiling, distillation, and storage at 4 °C until use [17]. 1-methyl-4-phenyl-1,2,3,6-tetrahydropyridine (MPTP) and 1-methyl-4-phenyl-1,2,3,6-tetrahydropyridine (MPP^+^) were purchased from Sigma-Aldrich (St. Louis, MO, USA). Levodopa was purchased from Merck (PHR1271; Supelco, Merck, Bellefonte, PA, USA), and an anti-tyrosine hydroxylase (TH) antibody (AB152; Merck Millipore, Burlington, MA, USA) was utilized for immunohistochemistry (IHC) and immunofluorescence (IF). All chemicals utilized in this study were of analytical or high-grade quality.

### 2.2. C. elegans Culture and Maintenance

Transgenic *C. elegans* strains BZ555 (Pdat-1::GFP) and NL5901 (Punc-54::α-synuclein::YFP) were obtained from the Caenorhabditis Genetics Center (CGC, University of Minnesota, Minneapolis, MN, USA) and maintained under standard conditions at 20 °C on nematode growth medium (NGM) agar plates seeded with *Escherichia coli* OP50 as a food source.

### 2.3. Fluorescence Imaging of Dopaminergic Neurodegeneration and α-Synuclein-YFP Fluorescence in C. elegans

To evaluate distinct PD-related phenotypes in *C. elegans*, BZ555 and NL5901 worms were used. BZ555 worms, which express GFP in dopaminergic neurons, were used to assess MPP^+^-induced dopaminergic neurodegeneration. NL5901 worms, which express human α-synuclein fused to yellow fluorescent protein (YFP) in body-wall muscle cells, were used to evaluate α-synuclein-YFP fluorescence as a surrogate readout of α-synuclein aggregation-associated proteotoxicity.

Synchronized L1-stage larvae of both strains were pretreated with deZP at concentrations of 0.25%, 0.5%, or 1% (*v*/*v*) in M9 buffer at 20 °C for 2 h. Following pretreatment, all experimental groups, except the normal control, were exposed to 4 mM MPP^+^, the active neurotoxic metabolite of MPTP, and incubated at 20 °C for 24 h. In BZ555 worms, MPP^+^ exposure was used to induce dopaminergic neurodegeneration, whereas in NL5901 worms, MPP^+^ exposure was used to increase α-synuclein-YFP aggregation-associated fluorescence under toxic stress conditions.

After treatment, worms were anesthetized with 4% sodium azide on a glass slide and immediately subjected to fluorescence microscopy using an Eclipse Ni-U microscope (Nikon Corporation, Tokyo, Japan). For fluorescence analysis, images from three worms per group that met technical quality criteria were used for quantitative analysis. The image selection criteria included clear focus, complete visibility of the worm body, absence of overlapping animals, and lack of fluorescence saturation. GFP fluorescence intensity in dopaminergic neurons and α-synuclein-YFP fluorescence in body-wall muscle cells were quantified using ImageJ software (version 1.54t; National Institutes of Health, Bethesda, MD, USA) and identical analytical parameters were applied across all groups. Fluorescence data were expressed as the mean ± standard deviation (SD) per individual worm. Fluorescence quantification was not performed in a blinded manner.

### 2.4. Mouse Experiments and Induction of the MPTP-Induced PD Model

Eight-week-old male C57BL/6N mice were purchased from Central Laboratory Animal Co., Ltd. (Seoul, Republic of Korea) and acclimatized for one week under a controlled environment with a temperature of 23 ± 1 °C, humidity of 60%, and a 12:12 h light/dark cycle with ad libitum access to standard food and water. Following the acclimatization, mice were randomly assigned to four groups (*n* = 8 per group): (1) control (saline-treated), (2) MPTP-alone, (3) MPTP + deZP, and (4) MPTP + levodopa. Behavioral assessments and histological analyses were performed by investigators who were aware of the treatment allocation. To reduce assessment bias, all behavioral tests and histological quantification were conducted using the same predefined criteria, apparatus settings, and analysis procedures across all groups.

The administration of deZP and levodopa was initiated 3 days prior to the first MPTP injection and maintained daily throughout the experiment, terminating one week after the final MPTP treatment (Figure 2A). deZP was administered intranasally under isoflurane anesthesia (Hana Pharm Co., Ltd., Hwa-Sung, Republic of Korea); a total volume of 20 μL/mouse (10 μL per nostril) was applied dropwise, corresponding to a dose of 5 mg/mouse based on the manufacturer’s labeled concentration of 0.25 g/mL. In this study, a single fixed dose was used; therefore, dose-dependent efficacy was not assessed. As detailed compositional data were not provided by the manufacturer, the chemical standardization and batch-specific profiling of the deZP preparation could not be determined in this study. As a positive control, levodopa (8 mg/kg, intraperitoneally) was administered at a dose previously shown to exert neuroprotective effects in MPTP-induced PD models. To establish the acute PD model, MPTP was dissolved in sterile saline and administered intraperitoneally four times on a single day at 2 h intervals at a dose of 20 mg/kg per injection, according to a standard protocol [27]. All animal experiments and care procedures were approved by the Institutional Animal Care and Use Committee of Woosuk University, Republic of Korea (approval no. WS-2025-15).

### 2.5. Behavior Tests

Before behavioral evaluation, all mice were individually identified and transferred from the animal care facility to the behavioral testing room.

The mice were allowed to acclimatize for 30 min to minimize stress associated with environmental changes and handling. Behavioral assessments were performed during the light phase under identical experimental conditions for all trials. The order of behavioral tests was standardized across all experimental groups to minimize variability. Rest periods were provided between consecutive trials according to each behavioral test protocol to reduce fatigue and stress and to ensure consistent motor performance. For the rota-rod and pole tests, mice underwent training sessions before the test day, as described below in the respective protocols.

#### 2.5.1. Rota-Rod Test

The rota-rod test, as previously reported [28], was used to assess motor coordination and balance. All experimental mice were placed on the rotating rod and allowed to stabilize before the acceleration sequence was initiated. Motor performance was evaluated under an acceleration protocol increasing from 5 rpm to 40 rpm over a period of 10 min. Each mouse underwent three independent trials, and the latency to fall was automatically recorded by the apparatus (HUAYON, Shenzhen, China). The mean latency value of the three trials was calculated and utilized for statistical analysis.

#### 2.5.2. Pole Test

The pole test was performed as previously described [29]. Briefly, each mouse was placed facing upward at the top of a vertical, rough-surfaced pole (0.8 cm in diameter and 60 cm in height). The total time for the mouse to turn around and completely descend from the top to the floor was recorded.

#### 2.5.3. Grip Strength Test

A grip strength meter (Model GS3; Bioseb, Chaville, France) was used to measure forelimb muscular strength. Each mouse was held by the base of the tail and allowed to grasp the metal grid with its forelimbs. The mouse was then gently pulled backward horizontally along its axis, and the maximum force (in grams) applied immediately before releasing the grid was recorded, as previously reported [30]. Each mouse completed three trials with a minimum inter-trial interval of 5 min. The mean value of the three consecutive measurements was used for statistical analysis.

### 2.6. Brain Tissue Collection and Preparation

Following the behavioral assessments, mice were transcardially perfused with 0.9% normal saline, followed by 4% paraformaldehyde (PFA) in PBS. Brains were carefully removed and post-fixed in the same fixing solution (4% PFA) at 4 °C for 24 h. For cryoprotection, the tissues were subsequently immersed in graded sucrose solutions (10%, 20%, and 30% *w*/*v* in PBS) for 24 h each at 4 °C until they sank. The cryoprotected brains were then embedded in FSC 22^®^ Clear Frozen Section Compound (Leica Biosystems, Richmond, IL, USA). Finally, the frozen brain tissues were sectioned coronally at a thickness of 30 µm using a cryostat (CM1860 cryostat; Leica Biosystems, Nussloch, Germany) and stored for further morphological analyses.

### 2.7. Immunohistochemical Analysis

The brain section was washed three times with PBS and incubated for 30 min at room temperature in a blocking solution containing 0.3% H_2_O_2_, 1% bovine serum albumin (BSA), 0.2% Triton X-100, and 1.5% normal goat serum. Following three washes with PBS, the sections were incubated overnight at 4 °C with TH primary antibody (1:500 dilution). After subsequent washing, the sections were incubated with the appropriate biotinylated secondary antibody (1:1000 dilution) for 1 h at room temperature. The signal amplification was performed using the VECTASTAIN^®^ Elite ABC-HRP Kit (PK-6100; Vector Laboratories, Burlingame, CA, USA) for 30 min. After washing with PBS, the immunoreactivity was visualized using 3,3′-diaminobenzidine (D4293; Sigma-Aldrich, St. Louis, MO, USA). Representative images of the immunostained sections were captured using an optical microscope (Leica DM 500; Leica, Wetzlar, Germany).

### 2.8. Immunofluorescence Analysis

Immunofluorescence staining was carried out in accordance with previously published protocols [31]. Slides containing brain sections were washed twice for 5 min and then permeabilized with 10 μg/mL Proteinase K (501-PK; Viagen Biotech, Los Angeles, CA, USA) for 5 min. After washing twice with PBS for 5 min, non-specific binding was blocked by incubating the sections with a solution containing 2% normal goat serum and 0.1% Triton X-100 in 0.1 M PBS for 1 h at room temperature. The sections were then incubated overnight at 4 °C with TH primary antibody (1:500 dilution). Following subsequent washes with PBS, the slides were incubated with the secondary antibody, Alexa Fluor™ 488 goat anti-rabbit IgG (A-11008; Invitrogen, Carlsbad, CA, USA), for 1 h at room temperature in the dark. After final washing, the slides were mounted with glass coverslips using an anti-fade fluorescence mounting medium (ab104135; Abcam, Cambridge, UK). Representative immunofluorescence images were captured using a fluorescence microscope (Eclipse Ni-U microscope; Nikon Corporation, Tokyo, Japan).

For histological quantification, anatomically matched coronal brain sections containing the SNpc or striatum were selected based on anatomical landmarks according to the mouse brain atlas. Three sections per group were analyzed. Images were acquired using identical microscope settings, including objective lens, exposure time, gain, light intensity, and resolution, across all experimental groups. Sections showing tissue damage, folding, incomplete staining, poor focus, or fluorescence saturation were excluded from analysis, and only sections in which the target anatomical region was clearly identifiable and tissue morphology was preserved were included. ROIs corresponding to the SNpc and striatum were manually delineated and analyzed using ImageJ software. TH-positive neurons in the SNpc were counted within the defined ROI, and TH immunoreactive intensity in the striatum was quantified as the mean signal intensity after background subtraction. The same threshold and analysis parameters were applied consistently to all images within each experiment.

### 2.9. Statistical Analysis

ImageJ software was used to quantify the number of TH^+^ neurons and the immunoreactive intensity of TH in the SNpc and striatum. GraphPad Prism 8 (GraphPad Software Inc., San Diego, CA, USA) was used to evaluate all behavioral and histological data. Quantitative data are expressed as mean ± SD. Before applying ANOVA, the assumptions of normality and homogeneity of variances were assessed using the Shapiro–Wilk test of residuals and the Brown–Forsythe test, respectively. Statistical differences among the experimental groups were analyzed using one-way analysis of variance (ANOVA), followed by Tukey’s post hoc test for multiple comparisons. Statistical significance was defined as *p* < 0.05. The following definitions were given to statistical symbols: # *p* < 0.05, ## *p* < 0.01, ### *p* < 0.001 compared to the control group; * *p* < 0.05, ** *p* < 0.01, *** *p* < 0.001 compared with the MPP^+^- or MPTP-alone group.

## 3. Results

### 3.1. deZP Attenuates MPP^+^-Induced Dopaminergic Neurodegeneration in C. elegans

**Figure 1:**
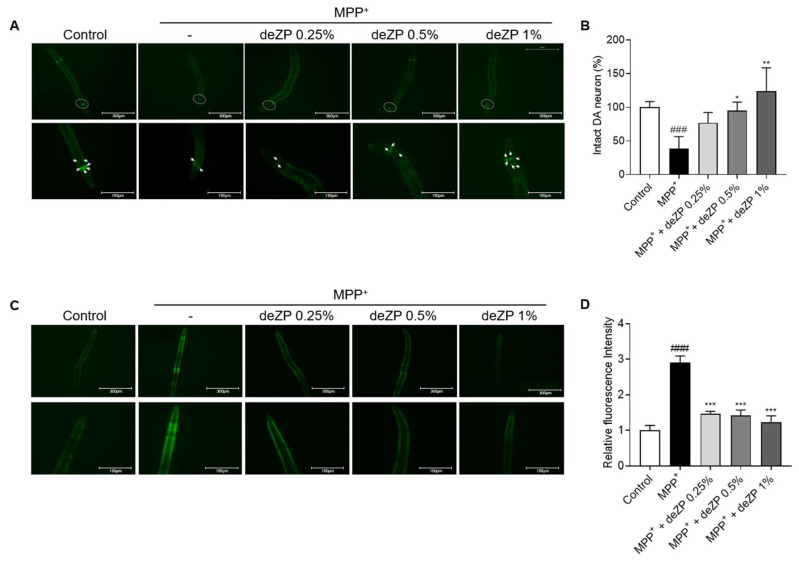
Protective effects of deZP against dopaminergic neurodegeneration and α-synuclein accumulation in *C. elegans* model of PD. (**A**) Representative fluorescence images of BZ555 worms (Pdat-1::GFP) following deZP pretreatment and MPP^+^ exposure. The dashed circle indicates the region enlarged in the lower panel. Arrows indicate intact DA cell bodies. (**B**) Quantification of intact dopaminergic (DA) neurons (%). (**C**) Representative fluorescence images of NL5901 worms (Punc-54::α-syn::YFP) following deZP pretreatment and MPP^+^ exposure. (**D**) Quantification of relative α-synuclein fluorescence intensity. Experimental groups consisted of normal control, MPP^+^ alone, MPP^+^ + deZP 0.25%, MPP^+^ + deZP 0.5%, and MPP^+^ + deZP 1%. Data are presented as mean ± SD of three independent experiments (*n* = 3). Statistical analysis was performed using one-way ANOVA followed by Tukey’s multiple comparison test. ### *p* < 0.001 vs. normal control; * *p* < 0.05, ** *p* < 0.01, *** *p* < 0.001 vs. MPP^+^-alone group.

To evaluate the protective effect of deZP against MPP^+^-induced dopaminergic neuronal degeneration, we used the BZ555 strain (Pdat-1::GFP), which selectively expresses GFP in dopaminergic neurons. In this model, dopaminergic neuronal damage was visually assessed by examining neuronal morphology and quantifying GFP fluorescence intensity. After MPP^+^ exposure, GFP fluorescence intensity in BZ555 worms was markedly reduced compared with the normal group (Figure 1A,B), indicating that MPP^+^ induced dopaminergic neuronal damage.

In contrast, deZP pretreatment partially restored the MPP^+^-induced loss of GFP fluorescence intensity at the effective concentrations tested. In particular, the administration of deZP at concentrations of 0.5% and 1% significantly increased GFP fluorescence intensity compared with the MPP^+^-alone group, accompanied by a remarkable preservation of dopaminergic neuronal morphology (Figure 1A,B). Conversely, the lowest concentration of deZP (0.25%) exerted no significant recovery effect against MPP^+^ toxicity. Quantitative analysis further confirmed that the percentage of intact dopaminergic neurons was significantly increased in the 0.5% and 1% deZP-treated groups. These results suggest that deZP, at effective concentrations, can attenuate MPP^+^-induced dopaminergic neuronal damage.

### 3.2. deZP Attenuates MPP^+^-Induced α-Synuclein-YFP Fluorescence in C. elegans

To determine whether deZP modulates α-synuclein aggregation-associated fluorescence, a PD-related proteotoxic phenotype, we used the transgenic *C. elegans* NL5901 strain. This strain expresses human α-synuclein fused with YFP (Punc-54::α-synuclein::YFP) in body wall muscle cells, allowing α-synuclein-YFP accumulation to be assessed based on YFP fluorescence intensity. Following MPP^+^ exposure, YFP fluorescence intensity in the NL5901 worms was markedly increased compared with the normal control group (Figure 1C,D), suggesting that MPP^+^ exposure increased α-synuclein-YFP aggregation-associated fluorescence under toxic stress conditions.

Notably, deZP treatment significantly attenuated the MPP^+^-induced increase in YFP fluorescence intensity. In particular, α-synuclein::YFP fluorescence signals were prominently diminished across all deZP-treated groups at concentrations of 0.25%, 0.5%, and 1% (Figure 1C,D). These findings indicate that deZP reduces α-synuclein-YFP aggregation-associated fluorescence, even at the lowest tested concentration (0.25%).

Taken together, these results demonstrate that deZP produces two complementary protective outcomes in *C. elegans*: attenuation of MPP^+^-induced dopaminergic neuronal damage in BZ555 worms and reduction in α-synuclein-YFP aggregation-associated fluorescence in NL5901 worms.

### 3.3. Intranasal deZP Administration Alleviates MPTP-Induced Motor Deficits in a Mouse Model of PD

To validate the protective effects of deZP observed in the *C. elegans* model at the in vivo level, we employed an MPTP-induced mouse model of PD. Following the establishment of the model, motor function was comprehensively evaluated using the rota-rod test, pole test, and grip strength test.

**Figure 2:**
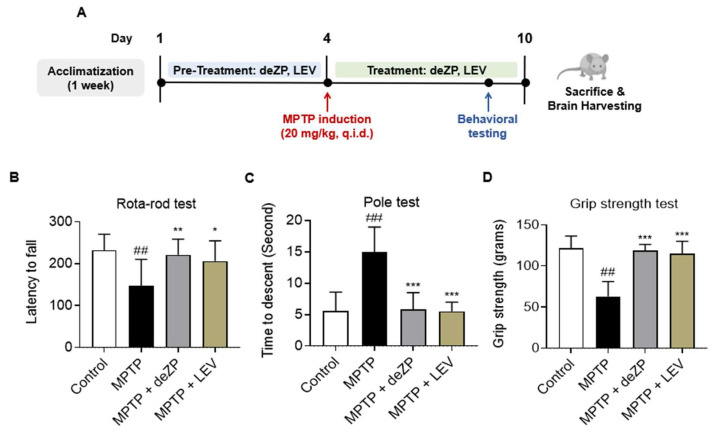
Neuroprotective effects of deZP on behavioral manifestations of MPTP-treated mice. (**A**) Schematic illustration of the experimental design and treatment timeline. (**B**) Rota-rod test (latency to fall), (**C**) Pole test (time to descend), and (**D**) Grip strength test. Experimental groups consisted of normal control, MPTP-alone, and MPTP + deZP-treated groups. Data were expressed as mean ± SD (*n* = 8 mice per group). Statistical analysis was performed using one-way ANOVA followed by Tukey’s post hoc test. ## *p* < 0.01 vs. normal control group; * *p* < 0.05, ** *p* < 0.01, *** *p* < 0.001, compared with the MPTP-alone group.

The rota-rod test was performed to assess motor coordination and balance. MPTP-treated mice showed a significantly reduced latency to fall compared with saline-treated control mice, indicating that MPTP induced motor coordination deficits (Figure 2B). In contrast, intranasal administration of deZP significantly prolonged the latency to fall compared with the MPTP-alone group, suggesting that deZP effectively rescued MPTP-induced impairment in motor performance. Notably, this improvement was comparable to that observed in the levodopa-treated positive control group under the present experimental conditions.

The pole test was subsequently utilized to evaluate locomotor activity, bradykinesia, and motor control. MPTP-treated mice required significantly more time to descend the pole than control mice, indicating severe impairment in movement initiation and agility following MPTP exposure (Figure 2C). However, intranasal deZP administration significantly shortened the descent time, counteracting the MPTP-induced bradykinesia. A similar improvement was observed in the levodopa-treated group under the present experimental conditions.

In the grip strength test, MPTP-treated mice showed a significant reduction in forelimb muscular strength, reflecting neuromuscular impairment (Figure 2D). In contrast, intranasal deZP administration significantly restored grip strength compared with the MPTP-alone group. A similar degree of recovery was also observed in the levodopa-treated group. Collectively, these results demonstrate that intranasal deZP broadly and effectively mitigates motor coordination deficits, locomotor impairment, and muscle weakness in the MPTP-induced mouse model of PD, producing improvements comparable to those observed in the levodopa-treated group under the present experimental conditions. 

### 3.4. Intranasal deZP Administration Protects Against MPTP-Induced Dopaminergic Neurodegeneration in the Substantia Nigra and Striatum

To determine whether the behavioral improvement induced by intranasal deZP was directly associated with dopaminergic neuroprotection, we performed IHC and IF analyses of TH in the SNpc and striatum of MPTP-induced PD mice. TH is a key enzyme involved in dopamine synthesis and is widely used as a representative marker for evaluating dopaminergic neurons and dopaminergic nerve fibers.

**Figure 3:**
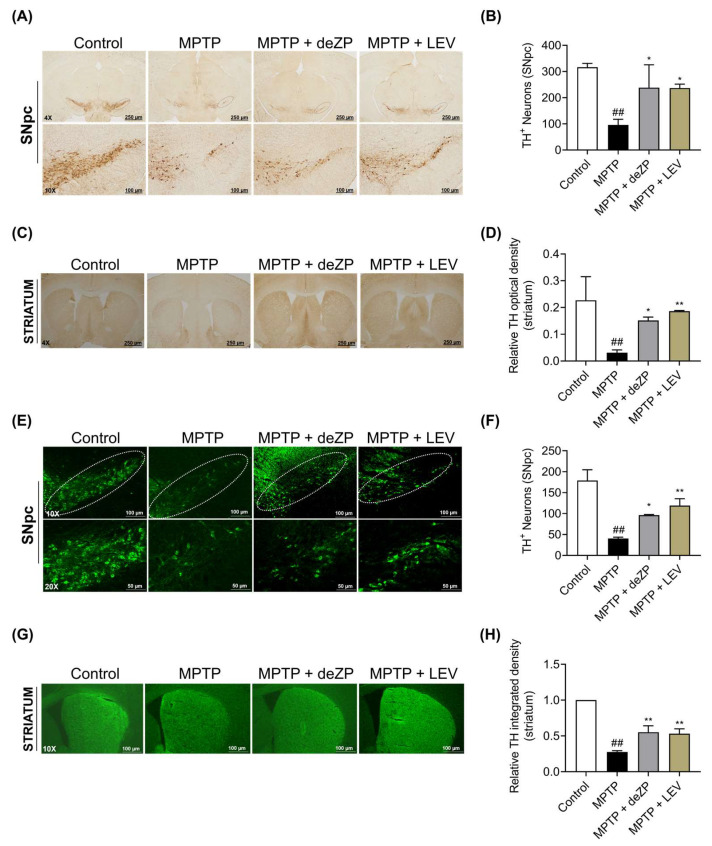
Comparative histological and immunofluorescence analyses of TH in the SNpc and striatum of MPTP-induced PD mice. (**A**) Representative IHC images showing TH^+^-immunoreactive neurons in the SNpc. The dashed circle indicates the region enlarged in the lower panel (magnification: 4× and 10×; scale bars: 250 μm and 100 μm). (**B**) Quantification of TH^+^ neuronal cell bodies in the SNpc via IHC. (**C**) Representative IHC images of TH^+^ dopaminergic fiber density in the striatum (magnification: 4×; scale bars: 250 μm). (**D**) Quantification of striatal TH optical density via IHC. (**E**) Representative IF images of TH^+^ neurons in the SNpc. The dashed circle indicates the region enlarged in the lower panel (magnification: 10× and 20×; scale bars: 100 μm and 50 μm). (**F**) Quantification of TH^+^ neurons in the SNpc via IF. (**G**) Representative IF images of TH^+^ immunoreactive fibers in the striatum (magnification: 10×; scale bars: 100 μm). (**H**) Quantification of striatal TH fluorescence intensity via IF. Experimental groups consisted of normal control, MPTP-alone, and MPTP + deZP-treated groups. Data are presented as mean ± SD (*n* = 3 mice per group). ## *p* < 0.01 vs. normal control group; * *p* < 0.05, ** *p* < 0.01 vs. MPTP-alone group.

First, TH IHC staining in the SNpc showed that the number of TH-positive neurons was markedly depleted in the MPTP-alone group compared with the control group (Figure 3A,B) confirming the successful induction of dopaminergic neurotoxicity. In contrast, intranasal deZP administration robustly rescued the loss of TH-positive neurons. A similar recovery pattern was observed in the levodopa-treated group under the present experimental conditions.

To investigate whether this neuroprotective effect extended to the projection site of these neurons, we analyzed TH immunoreactivity in the striatum.

The IHC analysis showed that TH-positive fiber density was clearly decreased in the MPTP-alone group compared with the control group (Figure 3C,D). This indicates that perikaryal loss in the SNpc was accompanied by terminal axonal degeneration in the striatum. Remarkably, intranasal deZP administration significantly restored the MPTP-induced reduction in striatal TH signals; a similar degree of recovery was observed in the levodopa-treated group under the present experimental conditions.

To strengthen these histological findings, we further validated the neuroprotective profile of deZP using IF analysis.

In agreement with the IHC data, TH IF staining in the SNpc demonstrated a severe loss of TH-positive neurons in the MPTP-alone group, which was significantly prevented by intranasal deZP administration (Figure 3E,F). Similarly, the MPTP-induced attenuation of TH-immunoreactive fibers in the striatum was substantially restored by deZP administration (Figure 3G,H).

Collectively, these results demonstrate that intranasal deZP administration attenuates motor coordination deficits, locomotor impairment, and muscle weakness in the MPTP-induced mouse model of PD, producing improvements comparable to those observed in the levodopa-treated group under the present experimental conditions. Consequently, these findings suggest that intranasal deZP may exert potential neuroprotective effects against PD-related dopaminergic neurodegeneration and motor dysfunction.

## 4. Discussion

In this study, we provide exploratory preclinical evidence that the intranasal administration of deZP, a preparation originally formulated for pharmacopuncture, attenuates MPP^+^- or MPTP-induced dopaminergic neuronal damage, α-synuclein aggregation, and motor deficits in *C. elegans* and mouse models of PD. PD is a representative neurodegenerative disorder histopathologically characterized by the progressive loss of dopaminergic neurons in the substantia nigra of the midbrain, which culminates in striatal dopamine depletion and subsequent motor dysfunction [3,32]. Given that the pathogenesis of PD involves highly interconnected and complex cascades—including α-synuclein proteotoxicity, mitochondrial impairment, oxidative stress, and chronic neuroinflammation [33,34,35]—the traditional “single-target, single-drug” approach may have limitations in addressing the multifactorial nature of PD. In this context, identifying naturally derived preparations with potential multi-target protective effects may be valuable for further preclinical investigation. Our findings suggest that intranasal deZP may represent a promising, albeit exploratory, preclinical candidate for further investigation in PD-related neurodegeneration.

To evaluate the initial in vivo efficacy of deZP, we utilized *C. elegans* transgenic models, which serve as an effective genetic platform to investigate evolutionarily conserved pathways of neurodegeneration [36,37]. *Z. piperitum* and its genus-derived secondary materials have previously been reported to possess neuroprotective properties; for instance, xanthoxyline suppresses dopaminergic neuronal degeneration via DAF-16-dependent signaling, while skimmianine modulates microglial activation and alleviates neural damage [13,14,15,16].

Building upon this phytochemical background, our bioassay using the BZ555 strain showed that deZP pretreatment attenuated the MPP^+^-induced reduction in dopaminergic neuronal GFP fluorescence. In the NL5901 strain, deZP treatment reduced α-synuclein-YFP aggregation-associated fluorescence. These findings suggest that deZP produces two complementary protective outcomes in *C. elegans*, although the molecular mechanisms underlying these phenotypic effects remain to be determined. These results provided a rationale for further evaluation of deZP in a mammalian PD model. These findings were further examined in a mammalian system using an MPTP-induced mouse model. MPTP selectively impairs dopaminergic neurons and is widely used to evaluate PD-related motor deficits [38,39]. In our behavioral assays, intranasal deZP administration improved MPTP-induced motor deficits, as evidenced by performance in the rota-rod, pole, and grip strength tests. These functional improvements were comparable to those observed in the levodopa-treated positive control group under the present experimental conditions. These findings support the potential protective effects of deZP in PD-related motor impairment in an experimental model.

These behavioral improvements were accompanied by histological preservation of the nigrostriatal dopaminergic pathway, a major anatomical substrate affected in PD. TH is widely used as a marker of dopaminergic neurons and fibers [40]. In the MPTP-alone group, TH-positive cell bodies in the SNpc and TH-positive axonal projections in the striatum were markedly reduced. Intranasal deZP administration attenuated these reductions, and similar patterns were observed in both IHC and IF analyses.

Because axonal degeneration can occur independently of or precede somatic degeneration in PD [41], the preservation of both striatal fibers and nigral cell bodies may provide a structural correlate for the observed motor improvements.

While the exact downstream molecular mechanisms underlying the effects of deZP remain to be fully elucidated, the observed protective effects may be partly explained by the bioactivities of volatile constituents previously identified in deZP. Previous phytochemical studies of deZP have identified terpinen-4-ol, d-limonene, citronellal, and geraniol as major volatile constituents [42,43]. However, the batch-specific chemical composition of the deZP preparation used in the present study was not independently re-analyzed, and the involvement of these individual compounds was not directly tested. Therefore, this interpretation should be considered hypothetical and literature-based.

Among these reported constituents, d-limonene has been suggested to exert neuroprotective and anti-inflammatory effects in neurodegenerative contexts, partly through modulation of NF-κB signaling and pro-inflammatory cytokine production [44]. Citronellal and geraniol have also been reported to reduce oxidative stress and mitochondrial dysfunction in experimental models by regulating antioxidant defense systems and cellular calcium homeostasis [45,46,47]. In addition, terpinen-4-ol has been associated with anti-inflammatory and antioxidant activities in previous experimental studies [18], suggesting that it may also contribute to the biological activity of deZP. Because PD pathogenesis involves interconnected processes such as oxidative stress, mitochondrial impairment, neuroinflammation, apoptosis, and α-synuclein aggregation, these previously reported bioactivities may provide a plausible basis for the protective phenotypes observed after deZP treatment. However, this mechanistic interpretation remains speculative. Future studies should directly evaluate the batch-specific chemical composition of deZP and examine molecular markers related to oxidative stress, neuroinflammation, mitochondrial function, NF-κB signaling, apoptosis, and α-synuclein aggregation to clarify the mechanisms underlying its protective effects.

Another methodological aspect of this study is the use of the intranasal route for deZP administration. In our previous study, we evaluated the systemic safety, biocompatibility, and stability of this formulation following intravenous injection [17]. Although systemic administration demonstrated the non-toxic profile of deZP, delivery of botanical extracts to the central nervous system may be limited by pharmacological barriers, including dilution in the systemic circulation and restricted permeability across the blood–brain barrier (BBB) [48]. In this context, intranasal delivery was selected as an exploratory non-invasive administration route that may favor CNS exposure, as previous studies have suggested that intranasally administered agents can access the brain through olfactory and trigeminal nerve-associated pathways and may partially bypass the BBB [49,50]. However, the present study did not directly demonstrate cerebral biodistribution, passage through the olfactory or trigeminal pathways, or increased central bioavailability of deZP after intranasal administration. Therefore, the behavioral and histological improvements observed in the MPTP-induced mouse model should be interpreted as supporting the potential usefulness of intranasal deZP administration in an experimental PD model, rather than as direct proof of enhanced CNS delivery.

Our findings demonstrate that intranasal administration enabled deZP to exert significant neuroprotective effects without causing detectable nasal mucosal toxicity, supporting the feasibility of this delivery strategy for targeting the central nervous system. However, the present study did not evaluate the pharmacokinetic profile or bioavailability of deZP following intranasal administration. Therefore, the extent of systemic absorption, nose-to-brain transport efficiency, and tissue distribution of its bioactive constituents remains to be determined. Future pharmacokinetic studies are warranted to clarify these parameters and to establish the relationship between intranasal exposure and neuroprotective efficacy. Although intranasal administration was selected in the present study to establish proof-of-concept neuroprotective efficacy with the aim of favoring brain delivery, this route does not preclude the nutritional relevance of *Z. piperitum* as an edible plant. Rather, our findings provide a biological rationale for further investigations into the potential of *Z. piperitum*-derived bioactive compounds as functional food ingredients or nutraceuticals. Future studies evaluating oral administration, gastrointestinal absorption, and long-term dietary supplementation will be necessary to determine whether the neuroprotective effects observed here can also be achieved through nutritionally relevant routes of administration.

The present study should be interpreted in light of several limitations. As this work was designed as an exploratory proof-of-concept study, only a single dose of deZP was evaluated in the mouse model, and the downstream molecular mechanisms underlying its protective effects were not directly examined. Therefore, future studies should include dose–response experiments and mechanistic analyses to define the optimal effective dose, therapeutic window, and signaling pathways involved in deZP-mediated neuroprotection. Although intranasal administration was selected as a non-invasive route that may favor CNS exposure, the present study did not directly evaluate pharmacokinetics, cerebral biodistribution, central bioavailability, or nose-to-brain transport efficiency. Route-specific pharmacokinetic and biodistribution studies are therefore needed to clarify the relationship between intranasal exposure and neuroprotective efficacy. In addition, the batch-specific chemical composition of the commercial deZP preparation used in this study was not independently re-analyzed. Previous phytochemical studies of deZP have identified terpinen-4-ol, d-limonene, citronellal, and geraniol as major volatile constituents; however, the exact chemical composition and relative abundance of these compounds in the batch used here could not be verified. Accordingly, the observed protective effects cannot be attributed to specific chemical constituents, and future studies should include independent chemical characterization, such as GC-MS or LC-MS analysis, to identify the active components responsible for these effects. Methodologically, mice were randomly assigned to the experimental groups, but behavioral assessments and histological analyses were not performed in a blinded manner. To reduce potential assessment bias, all behavioral and histological outcomes were evaluated using predefined and identical criteria across groups. Finally, because *Z. piperitum* is an edible plant, additional studies evaluating oral administration, gastrointestinal absorption, and long-term dietary supplementation will be necessary to determine whether the protective effects observed here can also be achieved through nutritionally relevant routes of administration.

## 5. Conclusions

Our study demonstrates that deZP attenuates MPP^+^-/MPTP-induced PD-related phenotypes in both *C. elegans* and mouse models. deZP treatment was associated with reduced α-synuclein-YFP aggregation-associated fluorescence in *C. elegans* and preservation of TH-positive dopaminergic neurons and fibers in the nigrostriatal pathway of MPTP-treated mice. In addition, intranasal administration was used as an exploratory non-invasive delivery route that may favor CNS exposure, although the present study did not directly demonstrate enhanced cerebral biodistribution or increased central bioavailability. Although further detailed investigations are warranted to precisely dissect the downstream molecular mechanisms, these findings support *Z. piperitum* as a promising edible source of bioactive phytochemicals with potential applications in the development of functional foods, nutraceuticals, and complementary strategies for neuroprotection.

The observed effects should be further confirmed through mechanistic analyses, dose–response studies, pharmacokinetic evaluation following intranasal administration, and additional PD models.

## Data Availability

The data supporting the findings of this study are available from the corresponding author upon reasonable request.

## Abbreviations

The following abbreviations are used in this manuscript:

ANOVA	Analysis of variance
BBB	Blood–brain barrier
BSA	Bovine serum albumin
CNS	Central nervous system
deZP	Distilled extract of *Zanthoxylum piperitum*
GFP	Green fluorescent protein
IF	Immunofluorescence
IHC	Immunohistochemistry
MPP^+^	1-Methyl-4-phenylpyridinium
MPTP	1-methyl-4-phenyl-1,2,3,6-tetrahydropyridine
NGM	Nematode growth media
PBS	Phosphate-buffered saline
PD	Parkinson’s disease
PFA	Paraformaldehyde
SD	Standard deviation
SNpc	Substantia nigra
TH	Tyrosine hydroxylase
YFP	Yellow fluorescent protein
